# Enhancing soot oxidation using microtextured surfaces

**DOI:** 10.1038/s41598-024-54320-5

**Published:** 2024-02-21

**Authors:** Oz Oren, Gordon McTaggart-Cowan, Sami Khan

**Affiliations:** School of Sustainable Energy Engineering, Simon Fraser University, Surrey, V3T 0N1 Canada

**Keywords:** Chemical engineering, Bioenergy

## Abstract

Biomass combustion provides energy needs for millions of people worldwide. However, soot accumulation on the combustors’ walls significantly reduces heat transfer efficiency. Herein, we demonstrate how microtextured surfaces minimize soot accumulation by enhancing soot oxidation. We investigate soot layers from the combustion of paraffin wax as a model for wood-based soot, and find that randomly microtextured glass obtained by sandblasting shows a 71% reduction in the time taken to oxidize 90% of surface soot coverage when compared to smooth glass at 530 °C. We also study grooved microtextures fabricated via laser ablation and find that grooves with widths between 15 and 50 µm enhance soot oxidation, while the expedited advantage is lost when the groove width is 85 µm. X-ray photoelectron spectroscopy validates the superior extent of soot removal on microtextures down to a sub-nanometer length-scale. The high density of sharp features such as peaks and edges on microtextures, and the conformality of the soot layer to the microtextures contribute to increased soot oxidation. We also demonstrate enhanced soot oxidation on microtextured stainless steel, the principal material of construction in biomass combustors. Microtextured surfaces that facilitate soot oxidation upon contact could significantly improve performance and longevity in various combustion applications.

## Introduction

Burning biomass provides space heating and cooking for many millions of people worldwide^1–3^. As biomass is composed primarily of 'current' carbon (i.e. carbon extracted from the atmosphere during a plant’s lifetime), the process is close to carbon-neutral^4^. The combustion of biomass typically emits significant amounts of carbonaceous particulate matter (PM; also referred to as soot)^5^. This biomass generated PM is one of the main contributors to indoor pollution^6,7^ and outdoor air pollution^8,9^ that leads to 8 million excess deaths per year^10^. Soot also contributes to climate change by changing cloud formation^11^ and absorbing solar radiation^12^.

Combustion systems are being developed that efficiently consume woody biomass and reduce harmful pollutant emissions. Soot precursors are formed during the combustion process and grow into long-chain aggregates in the post-flame regions^13,14^. Some of this soot is emitted, while the rest will build up on the exposed surfaces of the combustor and exhaust ducting^15^. Soot formation and deposition is particularly likely where the surfaces are cold, such as during start-up of a combustion process. This soot buildup acts as an insulator to reduce heat transfer, with a thermal conductivity on the order of 0.1 W/m.K^16^. Typical soot from woody biomass is composed of approximately 60% graphite and 40% polycyclic aromatic hydrocarbons (PAHs) along with traces of heavy metals and other ash^17^. This composition helps soot to adhere well to the surfaces typically found inside combustor systems, in addition to making it difficult to remove.

Soot accumulation on the inside walls of a combustor increases thermal resistance and decreases heat flux, reducing heat transfer from the combustion to the surrounding space. A thick soot layer will also potentially interfere with the combustion process itself and may lead to partial or complete blockages of the combustion exhaust system, with corresponding safety implications. Current methods for soot removal from biomass and fossil fuel combustors rely on active approaches using physical removal during maintenance cycles, and passive approaches using advanced catalytic materials. Soot layers are inherently superhydrophobic^18–21^ so it is difficult to remove them with water-based cleaning approaches, and often require the use of toxic organic solvents. Active approaches that have been studied include using supercritical CO_2_ as an environmentally-friendly solvent for dissolving carbonaceous deposits^22,23^ and laser surface cleaning^24^. Passive approaches employ soot oxidation on catalysts such as platinum, palladium, rhodium, ceria, iron, copper, nickel, silver^25^, and various combinations of these, including cobalt oxide nanoparticles coated with silver-cerium oxide^26^. Glass is also known to enhance soot oxidation^27–29^. These catalysts facilitate soot conversion into CO and CO_2_ by reducing the activation energy required to initiate the key exothermic oxidation reactions^30^ with some studies also reporting a reduced soot ignition temperature with the use of catalysts^31,32^. The potential to use surface microstructures, rather than chemical catalysis, to enhance soot oxidation has not been investigated.

There is a need to develop low-cost methods to reduce soot buildup inside biomass combustors that do not require constant cleaning or precious metal catalyst coatings of uncertain durability. Herein, we demonstrate a novel approach to reduce soot accumulation on surfaces using microtextures. The overall goal of this research is to quantify the effects of structured and unstructured microtextures on the rate of soot oxidation at a fixed temperature representative of the temperatures encountered in the inside surface of biomass combustors. The specific objectives of our study were to (1) assess whether roughening a surface through microtexturing can enhance soot oxidation compared to smooth surfaces; (2) evaluate the nature of the soot deposition on the microtextured surface; and (3) determine whether a residual soot layer is present on the microtextured surface after the soot appears to have oxidized.

## Methods

### Soot deposition setup

**Figure 1:**
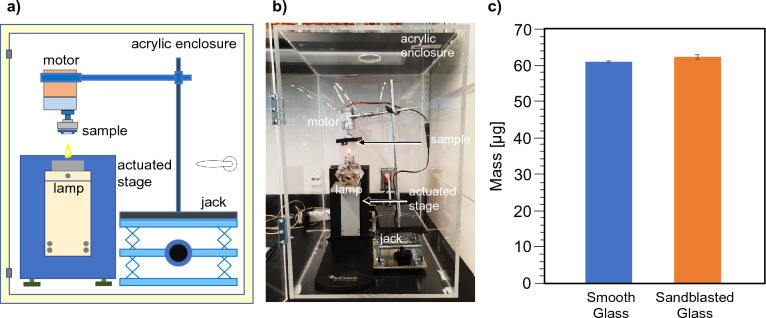
(**a**) Schematic showing systematic soot deposition on glass samples (**b**) Actual soot deposition setup used in this research (**c**) Comparison of mass of soot layer on smooth and sandblasted glass obtained after deposition for 10 s.

We designed a custom setup to deposit soot uniformly and consistently (Fig. 1a,b). We made four key components in this setup: a soot generator, a sample holder to position the sample above the soot generator, a motor to rotate the sample, and an actuated stage to provide precise height and timing. The motor rotated the samples at a constant 100 rpm to provide a uniform distribution of soot across all surfaces. We generated soot from a lamp filled with paraffin oil to minimize variations in soot chemistry arising from heterogeneity in woody biomass feedstock. The lamp also provided a repeatable flame height and shape to achieve uniform deposition of soot. We placed the lamp on an automated stage that was precisely manipulated in the z-direction (KSV Nima Dipcoater, computer controlled and modified for use as a stage). This automated process ensured that the substrates had a precise contact time of 1 s with the flame from the lamp, ensuring consistent mass of soot deposition between all samples (see Fig. 1c). Finally, we enclosed the setup in an acrylic enclosure to isolate the combustion system from the room environment. The integration of these four components in our system facilitated a systematic and consistent deposition of soot on the samples, minimizing human error and enhancing the accuracy of our investigations.

### Substrates

We selected glass as the substrate of interest to investigate soot oxidation. While steel is the most common material used inside biomass combustors^33^, we selected glass for this scientific study as it is relatively inert to both chemical and morphological changes at the high temperatures employed in this research, and it can be procured with very low surface roughness in the sub-micrometer length-scale. Furthermore, periodic microtextures could be created repeatably and consistently on glass from our excimer laser equipment. Glass is also often used in viewing window ports for residential wood burners.

We selected microscope glass slides (Ultident Scientific Inc., 76.2 mm by 25.4 mm with 1.0 mm thickness: see Supplementary Table S1 for glass composition). We considered the as-received slides to be “smooth glass”. We then created textured surfaces on the slides using two methods reported previously in literature to fabricate microtextures on glass: sandblasting^34^ and laser ablation^35^. We used a sandblaster (Mod-U-Blast S4826-3F) with glass beads of size 75–150 µm to generate a random surface texture. We also created repeating microstructured surfaces by cutting grooves into a smooth slide using an excimer laser with deep UV (IPG-280). We cut all the glass samples into 5 mm by 5 mm square coupons for soot oxidation experiments.

To assess the soot oxidation rate, it is first critical to have a consistent mass of soot deposited on the different substrates. We determined this by using a high-precision microbalance equipped with an autosampler (Perkin Elmer TGA 4000, precise to + /− 0.1 µg) to measure the change in substrate mass before and after soot deposition (See Fig. 1c). To collect enough soot to be able to measure the deposited mass, we exposed selected sandblasted and smooth samples to the flame for 10 s (n = 3 of each) using the systematic soot deposition setup described previously and used the autosampler in the microbalance to load the samples and measure the mass. As shown in Fig. 1c, the mass deposited on the smooth and sandblasted samples respectively was similar (2.13% difference), indicating a consistent mass deposition rate of soot from the paraffin lamp using our setup.

### Sample cleaning and soot deposition methodology

Prior to soot deposition, all samples were cleaned by sonicating in acetone and isopropanol for 5 min each, followed by thermal treatment at 530 °C in a box furnace. This procedure, adapted from cleaning methods for silicon wafers used in the semiconductor industry, ensured the glass surfaces were cleansed from environmental contaminants^36^. Soot layers were deposited on the clean glass coupons using the setup shown in Fig. 1a, b by exposing the samples to the flame for precisely 1 s from the top of the flame. Three samples were tested per experiment and statistical means and standard deviations were reported.

### Optical microscopy and image analysis to ascertain soot oxidation

After the soot was deposited, the samples were transferred to a Linkam heating stage and observed underneath a Zeiss AxioZoom V16 microscope connected to a recording camera (Nikon D7500). The temperature within the heating stage was increased from room temperature to the test experimental temperature (530 °C) with a ramp rate of 100 °C/min. This temperature was chosen based on the previously reported characteristic temperatures for soot oxidation on glass^29^. All experiments in this research were conducted at 530 °C, and the timer to track soot oxidation was started once the target temperature of 530 °C was reached.

The gathered soot oxidation videos were analyzed using ImageJ image analysis software, a method used in literature to visualize and interpret imaging data^37,38^. The soot deposition area varied between samples but the images for analysis were consistently obtained in the middle of the sample. Still images were extracted at 60 s intervals from the soot oxidation videos. The colour still images were converted to 8-bit grayscale, and a fixed intensity threshold of 130 was applied in ImageJ to define individual pixels as either covered with soot (intensity < threshold) and without soot (intensity > threshold). The total soot-covered and soot-free areas were then determined by adding the number of pixels and then multiplying by the individual area of each pixel. We then compared the areas of the sample with and without soot to the initial area for that sample. From this, we determined the change in soot oxidation area and consequently the percentage of soot oxidation over time based on the observed change in areas.

### Surface characterization

**Figure 2:**
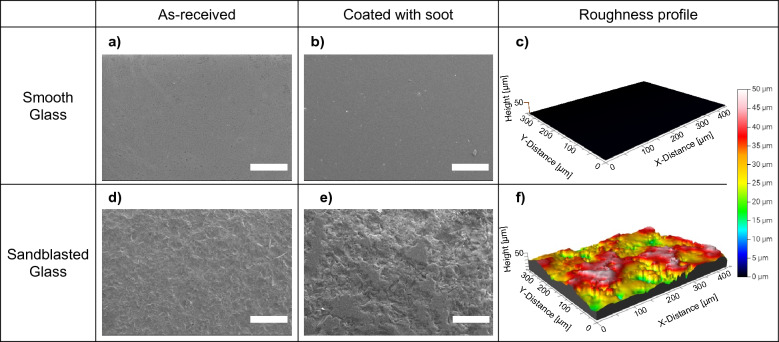
(**a**, **b**) SEM micrograph of smooth glass without and with a soot layer respectively (scale bar: 500 µm) (**c**) Optical profilometry image of smooth glass (**d**, **e**) SEM micrograph of sandblasted glass without and with a soot layer respectively (scale bar: 500 µm) showing conformal deposition of soot (**f**) Optical profilometry image of sandblasted glass.

X-ray Photoelectron Spectroscopy (XPS) was used to characterize the glass samples before and after soot oxidation (Thermofisher Scientific ESCALab 250XI). Mild sputtering with Ar^+^ ions (4000 eV large cluster ions) was performed in situ in the XPS to eliminate surface adventitious contaminants prior to analysis. Scanning electron microscopy (SEM) (Thermo Scientific Quattro ESEM) enabled the microscopic characterization of the glass surfaces with and without soot—see Fig. 2a,b for a smooth and Fig. 2d,e for a randomly roughened (sandblasted) surface. It can be seen from Fig. 2e that the soot deposition appears to result in a conformally deposited soot layer retaining the general roughened texture of the sandblasted surface. Optical profilometry (Bruker-Nano Contour GT-K) was used to compare and contrast the surface profile and surface roughness between smooth and sandblasted surfaces (Fig. 2c,f). The full surface roughness parameters obtained from optical profilometry are reported in Supplementary Table S2.

We also used XPS to compare soot chemistries emanating from different feedstocks: paraffin oil, candle wax, isopropanol and wood. As shown in Supplementary Table S3, the carbon content of the soot-covered surface was similar between all tested fuels, indicating that paraffin oil soot is a suitable surrogate for soot deposited by wood combustion. As the XPS measured to a depth of approximately 1 nm, and the soot layer thickness is on the order of micrometers, it is expected that the XPS results will reveal the nature of the soot layer on the surface and not the underlying glass substrate.

## Results and discussion

**Figure 3:**
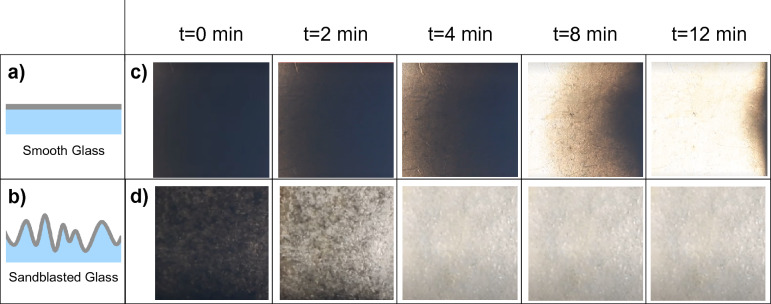
(**a**) schematic showing smooth glass covered with a uniform layer of soot (**b**) schematic showing sandblasted glass covered with a uniform, conformal layer of soot (**c**), (**d**) sequential optical microscopy images showing soot oxidation on smooth and sandblasted glass respectively. Sample dimensions in view in all optical microscopy images: 3.75 mm by 3.75 mm.

When subject to soot oxidation at 530 °C, the rate of soot oxidation was significantly expedited on sandblasted glass as compared to smooth glass (schematic in Fig. 3a,b) as shown in the sequential optical microscopy images in Fig. 3c,d and Supplementary Videos 1 and 2.

**Figure 4:**
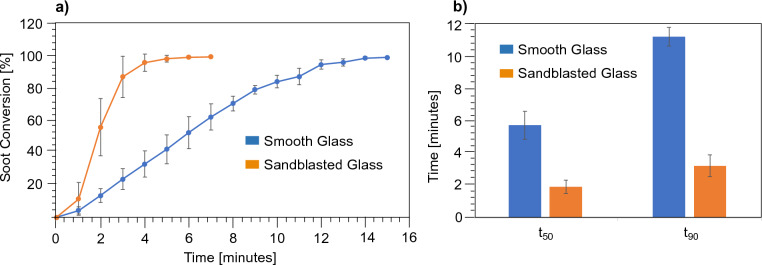
(**a**) Soot oxidation profiles for smooth and sandblasted glass—soot conversion against time. (**b**) Comparison of time taken to oxidize 50% of soot on the surface (t_50_) and 90% (t_90_) between smooth and sandblasted glass.

The soot oxidation profiles obtained from treating smooth and sandblasted glass samples at 530 °C are shown in Fig. 4a. The soot conversion percentage (Fig. 4a, y-axis) shown is the percentage of the total area from which soot has been oxidized based on image analysis. The optical microscopy images at t = 0 shown in Fig. 3c, d were fully covered with soot (i.e. 0% exposed glass) at the start of the oxidation phase. We represent the time it takes to oxidize 50% of soot as t_50_ and 90% of soot with t_90_, calculated using the percentage of the sample surface area where no soot was apparent. We observed a 66% reduction in t_50_ and 71% reduction in t_90_ using sandblasted glass when compared to the smooth surface (see Fig. 4b). The profiles shown in Fig. 4a also indicate a rapid initiation and propagation of the soot oxidation front using microtextured sandblasted surfaces as shown in Fig. 3 as well.

**Figure 5:**
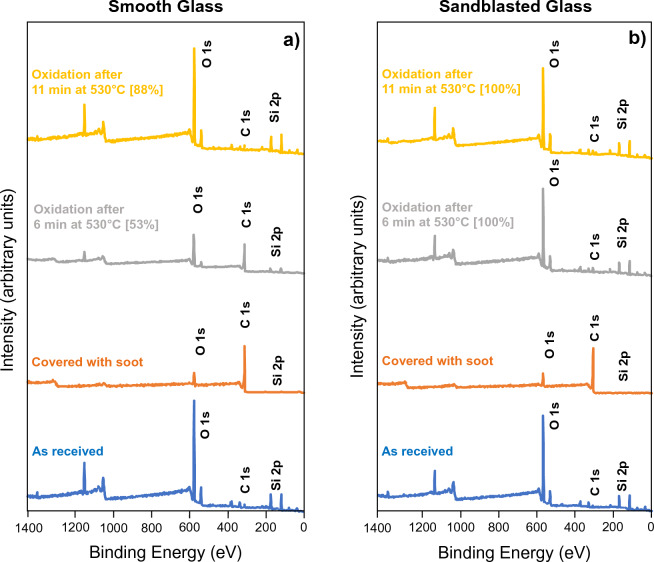
(**a**) X-ray photoelectron spectroscopy (XPS) spectra comparing surface chemistry profiles on smooth glass (**b**) X-ray photoelectron spectroscopy (XPS) spectra comparing surface chemistry profiles on sandblasted glass. Soot oxidation percentages determined from image analysis are shown in parentheses.

The surface chemistry before and after soot oxidation on both smooth and sandblasted glass was investigated using XPS. Survey spectra are shown in Fig. 5a,b and elemental surface composition as determined from high-resolution spectroscopy is shown in Table 1. The presence of Si2p and C1s peaks in the XPS spectra are correlated with soot oxidation performance: for samples covered with soot there is a stronger signal from C1s (285 eV), and for samples where soot is oxidized the underlying glass substrate becomes exposed, thereby showing a signal from Si2p (100 eV).

**Table 1:** Comparison of surface chemistry of samples tested in this research as obtained using X-ray Photoelectron Spectroscopy.

Surface	Surface Si content (%)	Surface O content (%)	Surface C content (%)
Smooth glass	28.1	66.3	5.7
Sandblasted glass	28.8	66.5	4.7
Smooth glass with soot	1.3	9.8	89.0
Sandblasted glass with soot	1.8	11.0	87.2
Smooth glass with soot, heated at 530 °C for 6 min	10.0	33.0	57.0
Sandblasted glass with soot, heated at 530 °C for 6 min	27.0	64.0	9.0
Smooth glass with soot, heated at 530 °C for 11 min	28.0	63.4	8.6
Sandblasted glass with soot, heated at 530 for 11 min	27.9	64.8	7.3

The smooth and sandblasted glass samples have similar surface chemistries as shown in Table 1. The composition of the soot layer is also comparable between smooth and sandblasted substrates, highlighting the consistency of our soot deposition method. The surface carbon content is similar to previously reported XPS analysis on candle soot^19^. For all samples, surface chemistry composition was reported based on C, Si and O surface coverage.

From visual analysis (Fig. 4a) we found that after 6 min the surface was free of soot on all tested sandblasted glass samples. XPS results validated the extent of soot oxidation: after 6 min, the surface chemistry profile of sandblasted glass was similar to the pristine sandblasted sample without soot. Since the XPS penetration depth was 1 nm, we can infer that the soot thickness is sub-nanometer after oxidation for 6 min, indicating superior soot removal on sandblasted surfaces. In contrast, the smooth glass still shows a high surface carbon content after 6 min. After 11 min both smooth and sandblasted glass have similar surface Si content indicating full removal of soot; this agrees with the results from the image analysis (Figs. 3 and 4) that show a highly reflective surface with little detectable soot after 11 min for both smooth and sandblasted samples.

Why do microtextured surfaces enhance the rate of soot oxidation? Oxidation of the soot layer broadly occurs in two key steps, initiation and propagation. Initiation occurs when there is sufficient oxygen present, and the temperature is sufficient to overcome the activation energy barrier for the key oxidation reactions^29,30^. Then, propagation occurs when the energy released from the exothermic soot oxidation reaction^30^ at the initiation site(s) heats the surrounding soot sufficiently that oxidation reactions continue to occur, creating a “front” that travels through the soot layer. Figure 3c shows that for smooth surfaces the soot oxidation initiates at the edges of the samples where the soot layer is likely thinner than the rest of the sample, and the oxidation front propagates from left to right as seen in the sequential images.

**Figure 6:**
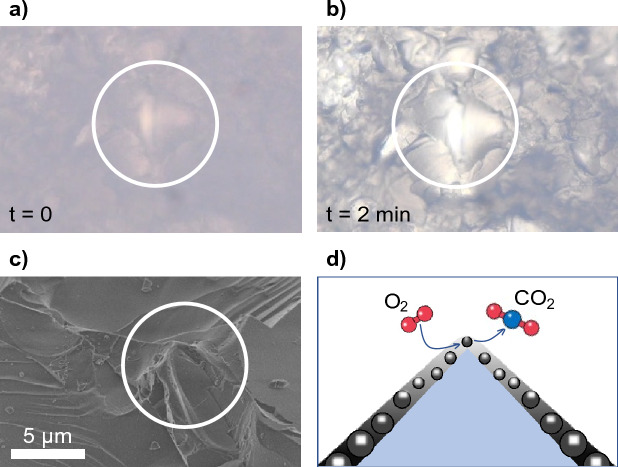
(**a**, **b**) Sequential optical microscopy images showing soot oxidation initiating on sharp peaks in the sandblasted glass (**c**) SEM micrograph image of the peaks in the sandblasted glass (**d**) Illustration of soot oxidation in peaks.

In contrast, we observed that for microtextured surfaces the oxidation initiated on the several peaks within the sandblasted surface and then propagated radially around each peak until the propagation fronts from neighbouring peaks merged (see Fig. 3d when interspersed specks appear at t = 2 min. as the glass substrate is revealed). Using a Zeiss AxioImager optical microscope (magnification up to 500x), we zoomed in to observe soot oxidation from a single peak in the sandblasted surface (Fig. 6a,b). We observed that the soot oxidation initiated from the peak and propagated along the slopes of the sharp feature shown (see SEM image in Fig. 6c). Unlike the smooth case where the initiation appears at the edge and propagates, in the rough case there are multiple internal sites where oxidation initiates. This collectively contributes to a faster oxidation rate.

**Figure 7:**
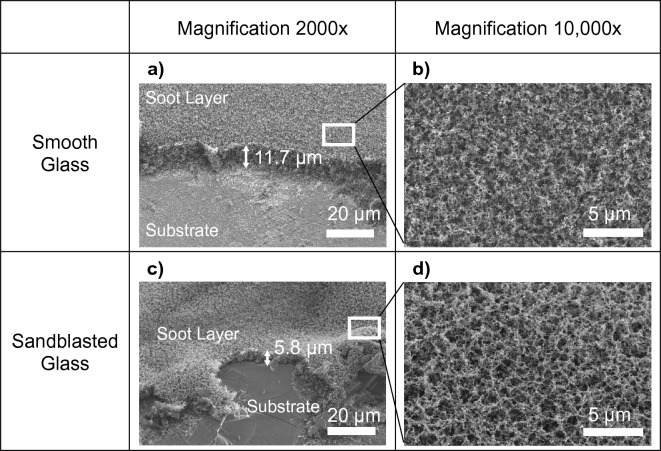
(**a**) SEM micrograph of the soot layer on smooth glass showing an average thickness of 11.7 µm. (**b**) Zoomed inset of A showing soot morphology on the smooth glass with dense soot agglomerates. (**c**) SEM micrograph of the soot layer on sandblasted glass showing an average thickness of 5.8 µm. (**d**) Zoomed inset of the peak from the soot-covered sandblasted glass in (**c**) with sparser soot agglomerates.

We hypothesize that a thicker soot layer is likely to oxidize slower than a thinner layer, and a densely packed soot layer is likelier to oxidize slower than one packed with lighter soot aggregates. SEM micrographs (see Fig. 7a,c) show a variation in height of the soot layer between the smooth and sandblasted samples − 11.72 ± 0.34 µm and 5.83 ± 1.07 µm respectively. This makes sense from the principle of conservation of mass as the sandblasted sample has a higher surface area than the smooth sample, thereby resulting in a thinner soot layer for the same soot deposition rate. This variation in the soot layer thickness between smooth and sandblasted samples likely contributed to the difference in soot oxidation rates seen previously.

Additionally, we also found that the soot layer on top of peaks consisted of sparsely packed soot aggregates (see SEM micrograph insets in Fig. 7b,d and schematic in Fig. 6d). This likely occurs because of the geometric constraint of a sharp peak resulting in the soot layer being internally “stretched”. Oxygen can permeate faster through sparsely packed soot aggregates thereby resulting in faster initiation of the oxidation reaction^39–41^. Furthermore, once the reaction initiates at the peaks it likely heats the local region from the enthalpy of combustion of soot, thereby rapidly propagating the reaction in the surrounding areas. All these factors likely contribute to enhanced soot oxidation on sandblasted surfaces.

### Grooved microtextures

**Figure 8:**
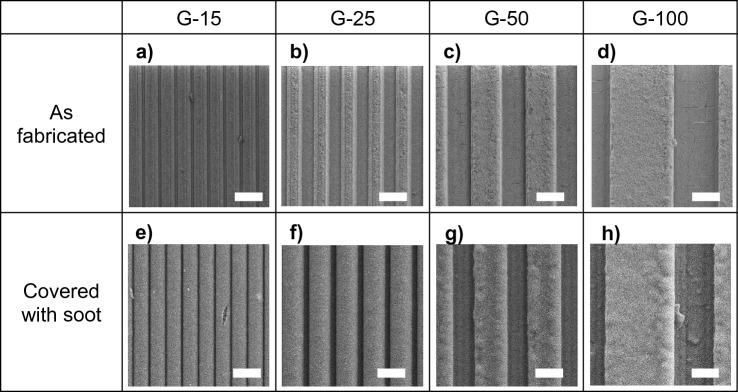
(**a**–**d**) SEM micrographs of systematic grooves prepared by laser texturing on glass with controlled groove width and spacing (**e**–**h**) SEM micrographs of the grooves in (**a**–**d**) covered with soot showing conformality. In all images scale bar = 50 µm.

The experiments above demonstrate that sandblasted textures expedite soot oxidation, and we also find that sharp peaks initiate the reaction faster. To investigate the role of microtextures more systematically, we designed grooves with controlled geometries using laser ablation (see Materials and Methods). Grooved samples were created using a square-beam laser of four spot sizes: 15 µm × 15 µm, 25 µm × 25 µm, 50 µm × 50 µm and 100 µm × 100 µm, and the samples were named G-15, G-25, G-50 and G-100 respectively (see Fig. 8a–d for SEM images). Trapezoid-shaped grooves and ridges were obtained as a result of laser ablation (see Supplementary Fig. S1 for schematic). The narrowest and widest part of the groove width were designated as *a*_*min*_ and *a*_*max*_ respectively, the spacing between the two grooves as *b*_*min*_ and *b*_*max*_, and the depth measured as the distance between the top of the ridge and the bottom of the groove as *c* (see Table 2 for dimensions measured using SEM). The soot deposition was mostly conformal as shown in Figs. 8e–h.

**Table 2:** Dimensions of the grooved samples prior to soot deposition.

Sample	Groove width, *a*_*min*_ [µm]	Groove width, *a*_*max*_ [µm]	Inter-groove spacing *b*_*min*_ [µm]	Inter-groove spacing *b*_*max*_ [µm]	Groove depth *c* [µm]	Ratio of *a*_*min*_/*a*_*max*_ α	Ratio of b_*min*_/*b*_*max*_ β	Number of sharp edges per mm^2^
G-15	5.7	15.7	14.0	23.8	5.0	0.36	0.59	67
G-25	23.4	32.0	20.2	28.9	8.1	0.73	0.70	38
G-50	43.2	48.6	52.3	56.9	14.2	0.89	0.92	20
G-100	82.5	85.2	116.3	117.6	23.8	0.97	0.99	10

**Figure 9:**
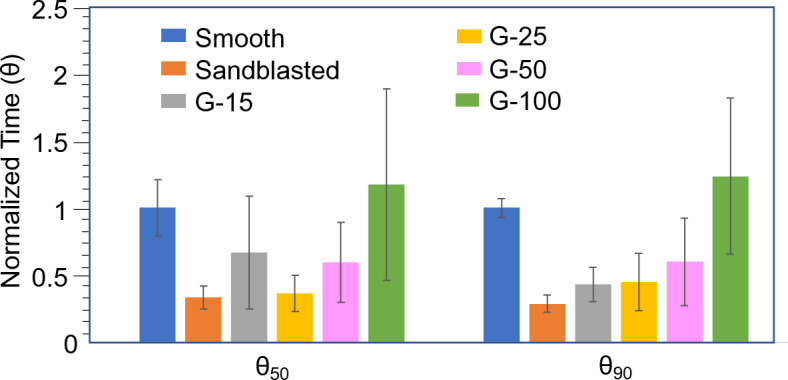
Comparison of time taken to oxidize 50% of soot on the surface (t_50_) and 90% (t_90_) by all microtextured glass surfaces tested in this research normalized by the soot oxidation time on smooth glass.

The relative impact of surface roughness on oxidation time can be compared between sandblasted and grooved microtextures using a normalized representation of time to oxidation, as shown in Fig. 9. The y-axis in Fig. 9 is the non-dimensionalized soot oxidation time, defined as $\theta = \frac{{{t_{textured}}}}{{{t_{smooth}}}}$. This was calculated by dividing the t_50_ and t_90_ on the rough textures by the corresponding t_50_ and t_90_ for the comparative smooth surface as determined from Fig. 3. The soot oxidation percentage to calculate θ was determined using the image analysis method previously described.

The parameter θ_50_ provides insights into the first half of the soot oxidation process consisting of initiation and propagation, while θ_90_ provides insights into the overall soot oxidation process with initiation, propagation and nearing termination. When θ < 1, it takes less time to oxidize soot on a rough surface as compared to that on a smooth surface. This effect was observed with θ_50_ and θ_90_ on the following textures: sandblasted surfaces and G-15, G-25 and G-50 grooved surfaces. Interestingly, G-100 fared worse than smooth as both θ_50_ and θ_90_ are greater than 1 as shown in Fig. 9.

**Figure 10:**
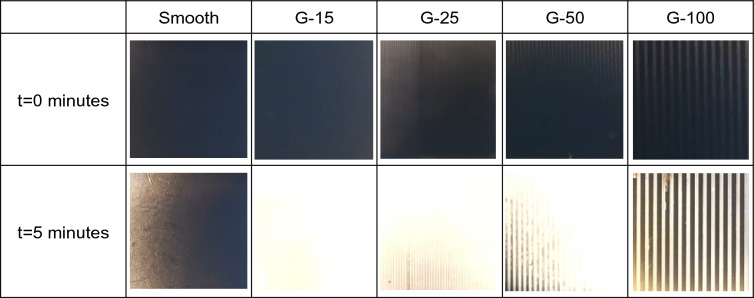
Sequential optical microscopy images at t = 0 and t = 5 min showing soot oxidation at 530 °C on smooth and laser-textured glass. Sample dimensions in view in all optical microscopy images: 3.75 mm by 3.75 mm.

Using optical microscopy (Fig. 10), we found that the soot within the grooves oxidizes before the soot on top of the ridges for all grooved samples tested. This effect is clearly visible in the G-100 case where the ridge tops still had unoxidized soot after 5 min while the soot deposited in the grooves appears to be fully oxidized (Fig. 10). We also observe that the initial soot layer within the grooves has a lighter shade than on the ridges; this is visible in the G-50 and G-100 cases in Fig. 10 at t = 0 min. This observation suggests that the soot layer within the grooves has a smaller thickness than the soot layer atop ridges, or it is more sparsely packed. If the grooves contain less soot, then by conservation of mass for a constant soot deposition rate (see Fig. 1c), the soot layer atop ridges should be thicker or denser in all grooved samples tested.

We observe from Fig. 9 that with the increase in groove width *a* from G-15 to G-100, there is an increase in θ_90._ Interestingly, while a similar trend with respect to θ_50_ is seen from G-25 to G-100_,_ the θ_50_ of G-15 is greater than that of G-25 and G-50 with larger error bars. These observations can be explained by analyzing the ratio between *a*_*min*_ and *a*_*max*_ (α), the ratio between *b*_*min*_ and *b*_*max*_ (β) and the density of sharp edges for all grooved samples shown in Table 2. We can see that the density of sharp edges decreases from 67 edges/mm^2^ to 10 edges/mm^2^ as the groove width *a* increases from sample G-15 to G-100 (see Supplementary Fig. S2 for calculation of edge density in G-100 found using the dimensions from Table 2). Contrastingly, the ratios α and β increase with groove width *a* in samples G-15 to G-100.

From experiments on the unstructured sandblasted surfaces, we know that soot oxidation initiates on sharp morphologies, so analogously it is likely that soot oxidation initiated on the sharp edges between ridges and grooves in samples G-15, G-25, G-50 and G-100. The ratios α and β represent the degree of skewness in the groove profile and ridge profile respectively, and hence the degree of sharpness of the ridge edges. For α = 1 and β = 1, both the groove and ridge profiles are rectangular with sharp ridge edges, and for α < 1 the profiles skew to a trapezoidal shape, resulting in blunter ridge edges. From Table 2 we observe that the ratio α for G-15 is less than half of that of G-25, while G-50 and G-100 are relatively more rectangular with their α close to 1. This can be explained by a microfabrication artefact resulting from the immediate redeposition of the material ablated from the groove by the laser along the nearby edges^35,42^—an effect that is most pronounced in G-15 which has the densest packing of grooves and ridges per square mm.

After soot deposition, the soot layer obfuscated the edges in G-15 leaving behind a thick layer on the ridges and virtually concealing the grooves as shown in Fig. 8e. This may explain why the θ_50_ of G-15 is larger than that of G-25 and G-50 despite G-15 having the higher edge density. The soot oxidation process is slowest to initiate with the obfuscated edges which are least sharp in G-15 as inferred from α and β. Instead, it is likely that the soot oxidation initiated within the grooves of G-15 which as described previously have the thinnest or sparsest packed soot layer compared to the ridges. The obfuscated, blunt edges and slow initiation at random locations within the grooves may also lead to larger experimental variability in the case of G-15, as demonstrated by the larger error bars for θ_50_ in Fig. 9. The competing effects of decreasing edge density and increasing edge sharpness with respect to the groove width likely resulted in a minima in θ_50_ at G-25.

While the soot oxidation is slow to initiate in G-15 as inferred by the higher value of θ_50_, the θ_90_ for G-15 is lowest among all grooved samples indicating that the soot oxidation rate significantly accelerates in G-15 once 50% of the soot layer has been oxidized. A possible explanation for this observation is the exothermic behaviour of the soot oxidation reaction^30^ and the thermally insulating nature of the soot layer^16^. As explained previously, the soot oxidation reaction likely initiates within the grooves of G-15. Surrounded by a thick insulating layer of soot, the localized substrate temperature in the buried grooves may exceed 530 °C from the enthalpy of the oxidation reaction. Given that soot oxidation rate increases as a function of temperature^41^ it is likely that this amplified localized temperature contributes to a speedier oxidation in G-15 as represented by the θ_90_.

Additionally, the higher degree of skewness in G-15 may also contribute to a greater extent of conformality of the soot layer to the sidewalls of the grooves. We know from experiments on sandblasted glass that conformality of the soot layer to the underlying substrate contributes to enhanced oxidation. The near-rectangular profiles of G-50 and G-100 may affect conformality along the grooves’ steep sidewalls slowing down the soot oxidation process in these samples in the longer run. A combination of all these geometric factors likely contribute to a direct correlation between the groove width and θ_90_ in all grooved samples as shown in Fig. 9.

Finally, for the smooth samples there was a well-defined oxidation front that progressed across the surface as shown in Fig. 3c. Any oxidation front moving along the upper surfaces of the ridges in the grooved samples would be interrupted by the grooves, slowing the overall oxidation. The low density of sharp features combined with a thicker soot layer atop ridges and the interruptions in the soot oxidation front likely contributed to both θ_50_ > 1 and θ_90_ > 1 for the G-100 sample. We infer from these results that in grooved samples there is a transitional density of sharp edges (between 20 edges/mm^2^ and 10 edges/mm^2^) whereby the advantage of grooves is lost compared to a smooth surface.

### Experiments on microtextured stainless steel

**Figure 11:**
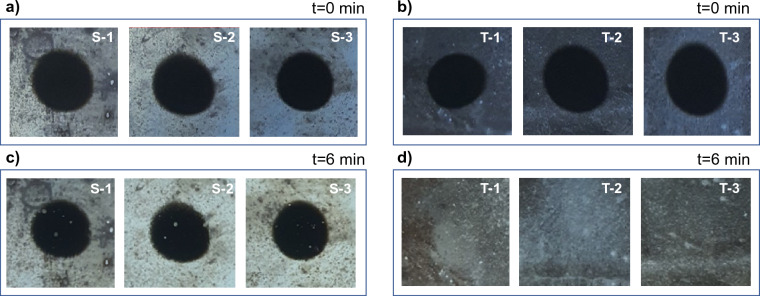
(**a**) three representative smooth stainless steel 420 (SS420) samples with deposited soot (designated S-1, S-2, S-3) (**b**) three representative sandblasted SS420 samples with deposited soot (designated T-1, T-2, T-3) (**c**) samples S-1, S-2, S-3 after 6 min at 530 °C showing soot still present (**d**) samples T-1, T-2, T-3 after 6 min at 530 °C showing complete soot removal. Sample dimensions in view in all images: 25.4 mm by 25.4 mm.

We observed soot oxidation on smooth and sandblasted stainless steel 420 samples. As-received stainless steel 420 (SS420, McMaster-Carr, 3.175 mm thickness) was cut into six identical 25.4 mm × 25.44 mm squares and cleaned using the same procedure used previously for glass samples. Three of these samples were microtextured using the same sandblasting process discussed previously. Soot was deposited on all samples using the systematic soot deposition apparatus described earlier, albeit the steel substrates were not rotated during soot deposition to simulate direct contact from the live combustion flame on the biomass combustor wall (see Fig. 11a,b). All six samples were placed in a furnace (Thermo Scientific Thermolyne Muffle Furnace F6010) for 6 min at 530 °C.

As shown in Fig. 11c, the as-received SS420 samples still had soot present after 6 min of thermal treatment at 530 °C, while all three representative sandblasted SS420 samples were devoid of soot as shown in Fig. 11d. Similar to glass, the presence of sharp peaks in the microtextured SS420 samples likely facilitated rapid initiation and propagation of soot oxidation. These features were absent in the as-received SS420 samples, thereby resulting in minimal soot oxidation as shown in Fig. 11c. These results demonstrate the versatility of microtextured surfaces on different types of materials.

## Conclusions

We systematically studied soot oxidation on microtextured surfaces and found that microtextured surfaces enhance the rate of soot oxidation. Randomly textured sandblasted glass surfaces reduced the time taken to oxidize 90% of surface soot coverage soot by 71% compared to a smooth glass surface with an equal mass of deposited soot. Similarly, grooved microtextured glass with widths between 15 and 50 µm also enhanced soot oxidation when compared to smooth glass. In contrast, grooved surfaces with 85 µm widths took longer to oxidize soot than smooth surfaces, indicating that under these conditions, there are optimal criteria for grooved microtextures to be effective for soot oxidation: width of grooves between 15 and 50 µm, and density of sharp edges between 20 edges/mm^2^ and 67 edges/mm^2^. We also demonstrated enhanced soot oxidation on microtextured stainless steel obtained by sandblasting, the main material of construction in biomass combustors. From our experimental observations we attributed the enhanced soot oxidation on microtextured surfaces to four key factors: (a) density of sharp edges and sharp peaks per unit area; (b) reduction in soot thickness offered by textures; (c) the change in aggregate density around sharp edges and sharp peaks; and (d) conformality of the soot layer to the underlying microtextures. In practical applications, we envision a faster, spontaneous removal of soot via oxidation from combustor surfaces covered with microtextures. In particular, unstructured microtextures resulting from sandblasting may perform best for soot oxidation: a randomly roughened surface is more likely to have sites that are ideally configured for initiating soot oxidation (high porosity soot agglomerates at the pinnacles of the structure) and to support propagation locally.

Reducing soot buildup by enhancing soot oxidation with microtexturing of the exposed surfaces offers potentially significant improvements in efficiency, improved safety, and reduced maintenance at a relatively low cost for biomass combustors used in home heating and cooking worldwide. Future work will explore mechanistic insights into the role of microtextures on soot oxidation, as well as extend the work to other materials such as metallic and ceramic substrates in a larger temperature range. Additional studies are necessary in future to understand the soot oxidation mechanisms on steel, and the effect of corrosion on microtextured surfaces alongside soot removal should also be investigated.

### Supplementary Information

Supplementary Information 1.

Supplementary Video 1.

Supplementary Video 2.

## Data Availability

The datasets generated during and/or analysed during the current study are available from the corresponding author on reasonable request.
