# Supplementary material for: Enhancing soot oxidation using microtextured surfaces
Source: Sci Rep. 2024 Feb 21;14:4247. doi: 10.1038/s41598-024-54320-5 (PMC10879092; doi:10.1038/s41598-024-54320-5)
Supplement: Supplementary file 1 — Supplementary Information 1. [file 41598_2024_54320_MOESM1_ESM.pdf]

Enhancing Soot Oxidation Using Microtextured Surfaces

Oz Oren[1], Gordon McTaggart-Cowan[1], Sami Khan[1*]

[1]School of Sustainable Energy Engineering, Simon Fraser University, Surrey V3T 0N1, Canada

Supplementary Information

Supplementary Tables:

Supplementary Table S1: Composition of glass as provided by the manufacturer

Component	Composition (Wt%)
SiO_2	70-73%
$Na_2O + K_2O$	13-15%
CaO	7-12%
Al_2O_3	1-2%
MgO	1-4.5%
SO_3	0-0.3%
Fe_2O_3	0.05-0.2%

Supplementary Table S2: Surface roughness parameters on smooth and sandblasted glass surfaces

		Developed area (Sdar) µm²	Projected area (Spar) µm²	Surface roughness (Sdar/Spar)	Peak height (Sp) µm	Valley depth (Sv) µm	Maximum peak to valley height (St) µm	Arithmetic mean height (Sa) µm	Root mean square height (Sq) µm
Smooth Glass	Sample 1	144900	144900	1	0.1637	0.1828	0.3465	0.07901	0.092
	Sample 2	146500	146500	1	0.3623	0.3584	0.7207	0.1725	0.1982
	Sample 3	146100	146100	1	0.177	0.1782	0.3552	0.08013	0.09146
	**Average**	**145833.3**	**145833.3**	**1**	**0.2343**	**0.2398**	**0.4741**	**0.1105**	**0.1272**
	**Standard Deviation**	**832.7**	**832.7**	**0**	**0.1110**	**0.1027**	**0.2136**	**0.0537**	**0.0615**
Sandblasted Glass	Sample 1	217300	144900	1.5	17.93	32.91	50.84	5.901	7.328
	Sample 2	264900	144900	1.83	40.08	59.02	99.1	15.38	18.6
	Sample 3	252500	144900	1.74	33.4	45.55	78.95	9.408	12.03
	**Average**	**244900**	**144900**	**1.69**	**30.47**	**45.83**	**76.30**	**10.23**	**12.65**
	**Standard Deviation**	**24693.3**	**0.0**	**0.17**	**11.36**	**13.06**	**24.24**	**4.80**	**5.66**

Supplementary Table S3: Comparison of surface chemistry of soot layers obtained from four feedstocks as determined using X-ray Photoelectron Spectroscopy

Surface	Surface Si content (%)	Surface O content (%)	Surface C content (%)
Paraffin wax soot (smooth glass)	1.3	9.8	89.0
Candle soot (smooth glass)	0	3.9	96.1
Alcohol soot (smooth glass)	0.4	5.2	94.4
Wood soot (smooth glass)	0.2	4.3	95.5

Supplementary Figures:

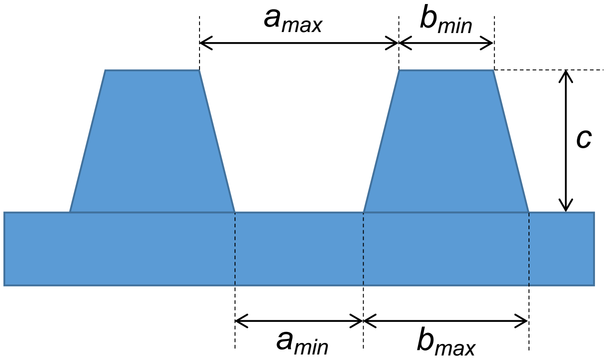

Supplementary Figure S1: Schematic showing the main dimensions in the grooved samples with:
a_{max}: edge-to-edge spacing between the ridge tops (also the maximum groove width)
a_{min}: minimum groove width at the base of the grooves
b_{max}: maximum ridge width at the base of the ridges
b_{min}: minimum ridge width at the top of the ridges
c: height of the ridges measured between the top of the ridge and the base

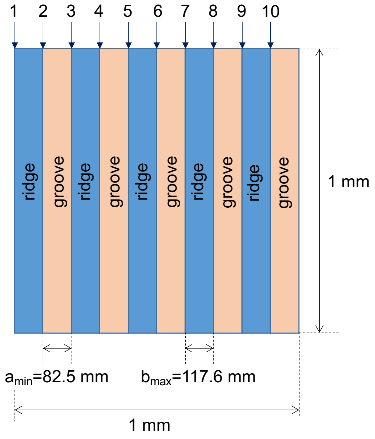

Supplementary Figure S2: Sample calculation to determine edge density in the G-100 grooved sample. The top corners of the trapezoids are characterized by sharp edges. Consequently, each trapezoid contributes two sharp edges. When examining a repeating unit within the groove samples (consisting of one ridge and one groove), it is observed that each repeating unit contributes two sharp edges. For example, to determine the number of sharp edges on G100 (100 µm grooves sample), the distance of a repeating unit, denoted by b_{max} and a_{min}, is 200 µm (117.6 + 82.5). Five repeating units are present in samples measuring 1000 mm (1000/200), yielding ten sharp edges (5 times 2 edges per repeating unit).

Supplementary Videos:

Supplementary Video 1: Soot oxidation on a smooth glass surface. Legend: Optical microscopy video showing soot oxidation on a smooth glass surface at 530°C, fast-forwarded 60 times. Sample dimension in view: 3.75 mm by 3.75 mm.

Supplementary Video 2: Soot oxidation on a sandblasted glass surface. Legend: Optical microscopy video showing soot oxidation on a sandblasted glass surface at 530°C, fast-forwarded 60 times. Sample dimension in view: 3.75 mm by 3.75 mm.
